# Integrating multi-omics, machine learning, and molecular dynamics simulations to identify glutamate metabolism-related biomarkers and drug candidates in rheumatoid arthritis

**DOI:** 10.3389/fmolb.2026.1834429

**Published:** 2026-04-29

**Authors:** Bingrui Zhu, Baoliang Li, Shuxu Zhang, Wenzhuo Qi, Zhou Mu, Peng Kong, Yingguang Han, Zhigang Shi

**Affiliations:** 1 Department of Minimally Invasive Orthopedics, The Affiliated Hospital of Shandong University of Traditional Chinese Medicine, Jinan, Shandong, China; 2 The First Clinical Medical School, Shandong University of Traditional Chinese Medicine, Jinan, Shandong, China

**Keywords:** biomarker, glutamate metabolism, machine learning, molecular dynamics simulation, rheumatoid arthritis, single-cell sequencing

## Abstract

**Background:**

Rheumatoid arthritis (RA) is a chronic autoimmune disorder marked by progressive joint destruction and functional impairment. Increasing data indicate that glutamate metabolism is critically involved in RA pathogenesis. This analysis aimed to identify glutamate metabolism-related biomarkers and potential RA therapeutics.

**Methods:**

Integrated analysis of data sourced from the GeneCards and Gene Expression Omnibus databases detected differentially expressed glutamate metabolism genes (DEGMGs). Functional enrichment analysis was implemented. Weighted gene co-expression network analysis and three machine learning algorithms were combined to detect potential RA biomarkers. Immune infiltration characteristics were evaluated via the CIBERSORT algorithm. Single-cell RNA sequencing delineated the cellular localization of biomarkers. Molecular docking and dynamics simulations screened for small-molecule drugs. Finally, quantitative real-time polymerase chain reaction and Western blot experiments in a fibroblast-like synoviocyte model verified the expression levels of detected biomarkers.

**Results:**

This analysis identified 322 DEGMGs. Enrichment analysis revealed their involvement in biological processes, including the tumor necrosis factor, phosphatidylinositol 3-kinase-Akt, and Janus kinase-signal transducer and activator of transcription signaling pathways. Machine learning algorithms ultimately pinpointed four core biomarkers. Combined molecular docking and dynamics simulations revealed favorable binding between azacitidine and the target proteins, characterized by high affinity and complex stability. *In vitro* experimental results were consistent with the bioinformatic predictions.

**Conclusion:**

This study identified four glutamate metabolism-related genes—CXCL10, ENTPD1, GPX3, and PSMB9—as potential biomarkers for RA. Azacitidine was also predicted as having therapeutic potential for RA. Together, these findings advance the understanding of RA pathogenesis and provide a novel theoretical foundation and candidate targets for its clinical diagnosis and targeted drug development.

## 1 Introduction

Rheumatoid arthritis (RA) constitutes a chronic autoimmune disorder primarily marked by sustained synovial inflammation and progressive joint destruction (Lee and Weinblatt, 2001). In accordance with the most recent Global Burden of Disease data, the global prevalence of RA reached approximately 17.9 million in 2021, with a higher prevalence observed in females (Ma et al., 2025). The disease exhibits a protracted and relapsing course. Early manifestations predominantly include joint swelling and pain, while advanced stages can lead to joint deformity and functional impairment (Huang et al., 2025). Additionally, RA possesses the potential to involve extra-articular organs (e.g., eyes, lungs, heart, and kidneys), thereby substantially influencing patient quality of life (Myasoedova et al., 2011). While significant advances in therapeutic strategies, including biologics, have provided new hope for RA patients in recent years, a considerable proportion of individuals exhibit an inadequate response or develop resistance to existing treatments (Zhang et al., 2020). Consequently, investigating diagnostic biomarkers and elucidating the pathogenic mechanisms of RA are crucial for early prevention and targeted therapy. This represents a current research priority.

As the most abundant non-essential amino acid in the human body, glutamate functions as a major excitatory neurotransmitter and a key metabolic intermediate (Miller et al., 2011). It exerts multifaceted regulatory roles in the pathogenesis of RA. Research indicates that glutamate participates in RA-associated inflammatory processes by activating the N-methyl-D-aspartate receptor (Flood et al., 2007; Gangadharan et al., 2011). Synovial fluid from RA individuals demonstrates notably elevated glutamate levels. This stimulates fibroblast-like synoviocyte (FLS) proliferation, thereby promoting inflammatory responses and joint destruction (Cowan et al., 2012). Secondly, glutamate is a critical intermediate in glutaminolysis. Upregulated expression of glutaminase 1 in RA synovial cells enhances the conversion of glutamine to glutamate (Kim et al., 2014). This glutamate subsequently contributes to alpha-ketoglutarate production, which enters the tricarboxylic acid cycle to supply cellular energy and biosynthetic precursors (Hanlon et al., 2022). It may also influence the differentiation balance between T helper 17 (Th17) cells and regulatory T cells (Tregs) through epigenetic regulation (Matias et al., 2021; Wen et al., 2025). Furthermore, glutamate is closely related to bone metabolism. Hajati et al. discovered a positive link between plasma glutamate levels and the degree of bone erosion in the temporomandibular joint of RA patients, particularly more pronounced in individuals with low inflammatory indices or aberrant sex hormone levels (Hajati et al., 2009). Nevertheless, current understanding of the interplay between glutamate metabolism and RA pathogenesis remains limited. Moreover, there is a clinical scarcity of RA biomarkers derived from glutamate metabolic pathways. Thus, a comprehensive and systematic analysis of glutamate metabolism-related genes (GMRGs) is particularly necessary.

The continuous advancement of bioinformatics technologies in recent years has provided powerful tools for deciphering the molecular mechanisms of diseases (Zhang et al., 2025). Leveraging RA transcriptomic data, this investigation systematically identifies potential RA biomarkers associated with glutamate metabolism through weighted gene co-expression network analysis (WGCNA), differential expression analysis, and machine learning approaches. Enrichment analysis elucidates the biological functions of core pathways. The CIBERSORT algorithm is adopted to evaluate immune infiltration features. External independent datasets and cellular models further verify the reliability and stability of candidate biomarkers. Concurrently, a single-cell transcriptomic dataset delineates the expression distribution of biomarkers across distinct cellular subpopulations within RA synovial tissue. Furthermore, molecular docking and dynamics simulations screen for small-molecule compounds with therapeutic potential. The goal of this study is to provide new insights into the pathogenesis of RA, optimize diagnostic strategies, and develop targeted therapies.

## 2 Methods and materials

### 2.1 Data acquisition and processing

Five RA-related microarray datasets (GSE55235, GSE55457, GSE12021, GSE1919, GSE89408) and one RA-related single-cell RNA sequencing dataset (GSE200815) were acquired from the Gene Expression Omnibus (GEO) (https://www.ncbi.nlm.nih.gov/geo/). GSE55235, GSE55457, GSE12021, and GSE1919 constituted the training set for subsequent model establishment. GSE89408 served as the validation set. Table 1 summarizes their basic characteristics. Unprocessed datasets were merged and standardized in R. Batch effects were corrected utilizing the “sva” R package. Additionally, 2,714 GMRGs were extracted from the GeneCards repository (https://www.genecards.org) employing a relevance metric cutoff surpassing five (Supplementary Table S1).

**TABLE 1:** Details of gene expression dataset.

Dataset	Platform	Tissue	Rheumatoid arthritis	Control	Experiment type	Class
GSE55235	GPL96	Synovium	10	10	Microarray	Training set
GSE55457	GPL96	Synovium	13	10	Microarray	Training set
GSE12021	GPL96	Synovium	12	9	Microarray	Training set
GSE1919	GPL91	Synovium	5	5	Microarray	Training set
GSE89408	GPL11154	Synovium	152	28	High-throughput sequencing	Validation set

### 2.2 Differential expression analysis

Assessment of differential expression profiles across RA and control groups was implemented via the “limma” R package. Thresholds were set at |log2 fold change (log2FC)| ≥ 0.585 and a false discovery rate (FDR) < 0.05. Volcano plots and heatmaps were generated to visualize the identified differentially expressed genes (DEGs) and their expression patterns. Then, the obtained DEGs were intersected with the GMRGs, yielding differentially expressed glutamate metabolism genes (DEGMGs).

### 2.3 Functional enrichment analyses

To elucidate the core biological processes, associated signaling cascades, and potential regulatory networks of the DEGMGs, functional enrichment analysis was conducted using the “clusterProfiler” package based on the “org.Hs.eg.db” annotation database. This analysis encompassed Kyoto Encyclopedia of Genes and Genomes (KEGG) pathway enrichment and the three core Gene Ontology (GO) sub-ontologies: cellular component (CC), molecular function (MF), and biological process (BP). An FDR <0.05 served as the threshold for significant enrichment.

### 2.4 WGCNA

WGCNA was conducted on the training set. Sample outliers were initially checked via hierarchical clustering. An appropriate soft-thresholding power (β) was chosen to create a scale-free network. The adjacency matrix was computed and transformed into a topological overlap matrix (TOM). A gene clustering tree was built based on TOM distance. The dynamic tree cut algorithm partitioned initial modules, requiring a minimum module size ≥200 genes. Modules with highly similar module eigengenes (MEs) (distance <0.3) were merged. Pearson correlations and p-values between each merged module’s ME and the RA trait were computed. A module-trait relationship heatmap was plotted to identify key modules most notably related to RA.

### 2.5 Machine learning algorithms

A combination of three feature selection methods—support vector machine-recursive feature elimination (SVM-RFE), random forest (RF), and least absolute shrinkage and selection operator (LASSO) regression—was applied to refine the selection of candidate core genes. LASSO regression with 5-fold cross-validation was implemented utilizing the “glmnet” package. The optimal regularization parameter (lambda.min) was determined via minimum cross-validation error. Genes with non-zero coefficients were retained as the LASSO feature set. SVM-RFE recursively eliminated the least important features to enhance model performance. Subsets from 2 to 40 genes were evaluated sequentially, with the optimal gene number determined by the smallest root mean square error. The RF algorithm, an ensemble learning method, constructed a model with 1000 trees. The out-of-bag error rate versus the number of trees was plotted to determine the optimal tree count. A new model was built using this count, and genes with a mean decrease in Gini >1 and ranked in the top 10 for importance were selected. Finally, the “ggvenn” package identified the intersecting genes from the three algorithms, designating them as core genes.

### 2.6 Diagnostic potential and expression analysis of hub genes

To validate the diagnostic accuracy of the machine learning-derived hub genes, receiver operating characteristic (ROC) analysis was carried out employing the “pROC” package. The area under the curve (AUC) quantified diagnostic efficacy. Boxplots, generated with “ggplot2”, displayed expression level differences of candidate biomarkers between the training and validation datasets.

### 2.7 Gene set enrichment analysis (GSEA) of hub genes

GSEA is an algorithm that evaluates the enrichment of a predefined gene set within a ranked gene list. Utilizing R packages including “limma”, “org.Hs.eg.db”, “clusterProfiler”, and “enrichplot”, a continuous ranked gene list was constructed based on Pearson correlation coefficients between hub genes and the genome-wide expression profile. This list was subjected to GSEA (KEGG and GO) to uncover potentially regulated biological processes, with significance set at FDR <0.05.

### 2.8 Immune infiltration analysis

The CIBERSORT deconvolution algorithm analyzed the gene expression data to estimate the relative abundance of various immune cell types within specimens. Wilcoxon tests compared immune cell differences between groups. Spearman’s rank correlation analysis explored correlations of hub gene expression with immune cell infiltration in RA. The objective was to clarify potential links between gene expression and the immune microenvironment.

### 2.9 Single-cell RNA sequencing

The “Seurat” R package analyzed the GSE200815 dataset to construct a single-cell expression profile of RA synovial tissue. Quality control filtered cells with nFeature_RNA >200, nCount_RNA >500, and percentage of mitochondrial genes <18%. The SCTransform algorithm performed normalization, correcting for sequencing depth variation via regularized negative binomial regression while preserving highly variable genes. Principal component analysis (PCA) reduced dimensionality. The ElbowPlot assessed principal component contributions, guiding the selection of the first 20 principal components for subsequent analyses. The Harmony algorithm corrected batch effects. Cell clustering employed a resolution parameter of 0.6. The “FindAllMarkers” algorithm identified signature genes for individual clusters. Cell types were annotated employing the CellMarker database (http://xteam.xbio.top/CellMarker/) and relevant literature. The resulting annotations were visualized via Uniform Manifold Approximation and Projection. The “FeaturePlot” function visualized the expression and distribution of key biomarkers across cell types. Additionally, the “CellChat” package investigated intercellular communication networks within the RA synovial tissue microenvironment.

### 2.10 Connectivity map (CMap) drug prediction

The CMap (https://clue.io) database contains gene expression profiles for connecting gene signatures, disease states, and small molecules. DEGMGs were divided into up- and downregulated groups per expression trend and submitted to CMap to predict small molecules with therapeutic potential for RA. According to the CMap scoring metric (−100 to 100), a negative connectivity score indicates that the compound’s expression profile inversely correlates with the disease signature, suggesting potential to reverse the pathological state. The top 10 compounds with the most negative connectivity scores were selected as candidate drugs for RA.

### 2.11 Molecular docking

Molecular docking experiments evaluated the potential therapeutic activity of candidate compounds. The three-dimensional structures of the primary active ingredients were procured from the PubChem repository (https://pubchem.ncbi.nlm.nih.gov/). The crystal structures of the target proteins CXCL10, GPX3, and PSMB9 were obtained from the Protein Data Bank (PDB; http://www.rcsb.org/). ENTPD1 was excluded due to the absence of an experimentally resolved structure. Water molecules, heteroatoms, and original ligands were removed using PyMOL, saving the structures in PDB format. The GetBox Plugin determined the binding pocket center and dimensions. AutoDock Tools 1.5.6 converted the protein and ligand files to PDBQT format. Molecular docking was carried out via AutoDock Vina 1.1.2, with a docking score < −5.0 kcal/mol generally indicating favorable binding activity. Finally, PyMOL 2.6.0 was used to visualize the docking results.

### 2.12 Molecular dynamics simulation

Gromacs 2022 conducted molecular dynamics simulations. The AMBER14SB force field parameterized the receptor protein, while the General AMBER Force Field (GAFF2) described the ligand. The system was hydrated within a TIP3P aqueous cuboid with a 1 nm edge length (Mark and Nilsson, 2001). Counterions were added to ensure electroneutrality. The particle mesh Ewald method handled long-range electrostatic interactions with a 1 nm cutoff. The SHAKE algorithm constrained all bonds. The Verlet leapfrog algorithm performed integration with a 1 fs time step. Before production simulation, the system underwent energy minimization involving 3,000 steps of steepest descent followed by 2,000 steps of conjugate gradients. The simulation ran for 100 ns under an NPT ensemble at 310 K. Trajectory analysis utilized built-in GROMACS tools. System stability and dynamic characteristics were evaluated by computing the number of hydrogen bonds, root mean square fluctuation (RMSF), radius of gyration (Rg), root mean square deviation (RMSD), and solvent accessible surface area (SASA).

### 2.13 Cell culture

SHC8713 (human RA FLSs) and SHC8719 (human synovial fibroblasts) were purchased from Shanghai Chunmai Biotechnology Co., Ltd. Cells were routinely cultured at 37 °C in DMEM/F12 medium (11320033, Gibco), supplemented with 10% fetal bovine serum (abs972, Absin) and 1% penicillin-streptomycin (U31-301C, YOBIBIO), in a 5% CO_2_ incubator. Upon reaching 80%–90% confluence, cells were passaged at a 1:3 ratio using 0.25% trypsin. Cells were passaged three times weekly, and those in the logarithmic growth phase were utilized for subsequent experiments.

### 2.14 Quantitative real-time polymerase chain reaction (qRT-PCR)

Total RNA was extracted from cells using TRIzol reagent (15596018, Invitrogen, United States). One microgram of purified RNA was quantified and reverse transcribed using the ReverTraAce qPCR RT Kit (P112-03, Vazyme, China). Quantification of mRNA expression was conducted via the 2^^–ΔCT^ method, normalized to GAPDH. Primer sequences are provided in Table 2.

**TABLE 2:** Primer information.

Gene	Forward primer (5′–3′)	Reverse primer (3′–5′)
CXCL10	CCCACGTGTTGAGATCATTG	TCCATCACAGCACCGGG
ENTPD1	AGGTGCCTATGGCTGGATTAC	CCAAAGCTCCAAAGGTTTCCT
GPX3	GGGGATGTCAATGGAGAGAA	TTCATGGGTTCCCAGAAGAG
PSMB9	TTGTGATGGGTTCTGATTCCCG	CCATGTCGGCCACGGCTTGGG
GAPDH	CGAAATCCCATCACCATCTTCCAGG	GAGCCCCAGCCTTCTCCATG

### 2.15 Western blot (WB)

Total cellular protein was isolated using RIPA lysis buffer (CBW0011, COBIO), with incubation on ice for 30 min, followed by centrifugation at 12,000 rpm for 10 min at 4 °C. The supernatant was quantified using a BCA protein assay kit (CBW0020, COBIO), mixed with 5× loading buffer (CBW0029, COBIO) at a 4:1 ratio, and denatured by boiling for 15 min. Proteins were separated by SDS-PAGE (75 V for stacking gel, 120 V for separating gel) and transferred onto methanol-activated PVDF membranes (IPVH00010, Millipore) at 300 mA constant current for 40 min. After blocking with 5% BSA (V900933, Merck) for 1 h at room temperature, membranes were incubated with primary antibodies [anti-GAPDH (60004-1-Ig, Proteintech), anti-CXCL10 (YT2379, Immunoway), anti-ENTPD1 (DF4031, Affinity), anti-PSMB9 (14544-1-AP, Immunoway), anti-GPX3 (YN1986, Immunoway)] overnight at 4 °C. Subsequent to TBST (G0004, Servicebio) washes, membranes were cultured with secondary antibodies at ambient temperature for 30 min and washed again. Protein bands were visualized using an enhanced chemiluminescence kit (P0018M, Beyotime) and detected with a ChemiScope 6100 system (CLiNX, China). Band intensity was quantified using AlphaEaseFC software.

### 2.16 Statistical analysis

Bioinformatics analyses and visualization were implemented in R (v4.5.1). Data from *in vitro* experiments were processed and graphed using GraphPad Prism (v10.0). All data were reported as mean ± standard deviation. Intergroup statistical differences were assessed using the Wilcoxon rank-sum test or the unpaired t-test, as appropriate. Correlation analyses were conducted using either the Pearson correlation analysis or Spearman’s rank correlation test. In addition, the Benjamini–Hochberg correction method was applied to control the FDR. A p-value below 0.05 was considered statistically significant.

## 3 Results

### 3.1 Detection of DEGs

**FIGURE 1:**
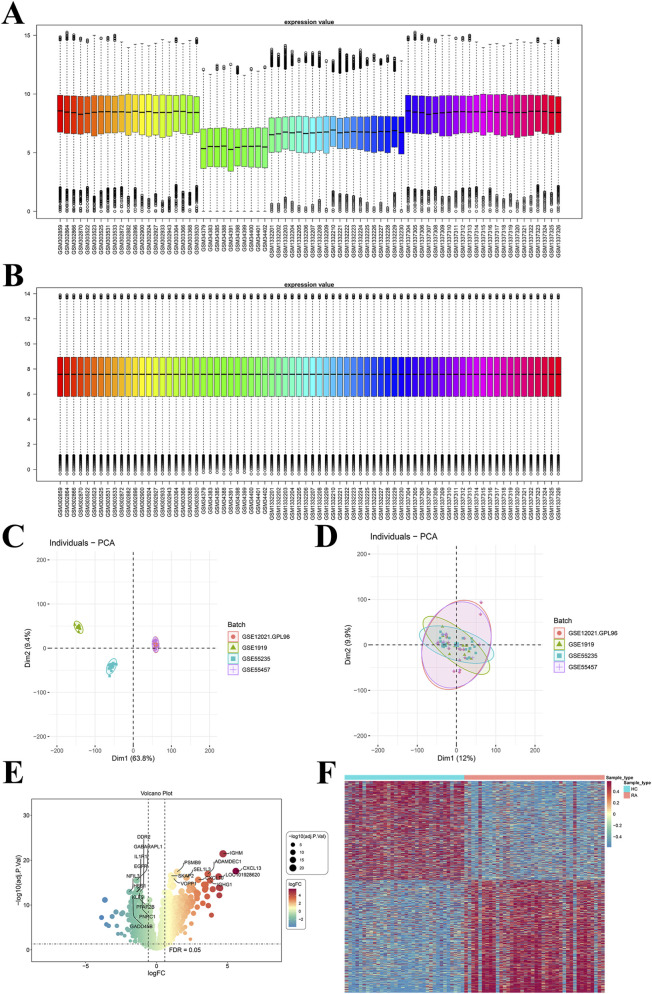
Differentially expressed gene Analysis. **(A)** Box plot of GEO dataset distribution before batch processing; **(B)** Box plot of GEO dataset distribution after batch processing; **(C)** PCA plot before batch processing; **(D)** PCA plot after batch processing; **(E)** Volcano plot of DEGs; **(F)** Heatmap of DEGs.

Initial data standardization checks revealed dispersed distribution and significant batch effects across datasets (Figures 1A,C). After batch effect correction, subsequent boxplots and PCA plots demonstrated effective removal of these effects (Figures 1B,D). Differential expression analysis identified 1,290 DEGs, comprising 685 upregulated and 605 downregulated genes. A volcano plot visualized the top 10 down- and upregulated DEGs (Figure 1E). A heatmap displayed divergent expression profiles across the two sample cohorts (Figure 1F). Intersecting the DEGs with GMRGs yielded 322 DEGMGs.

### 3.2 Functional pathway enrichment of DEGMGs

**FIGURE 2:**
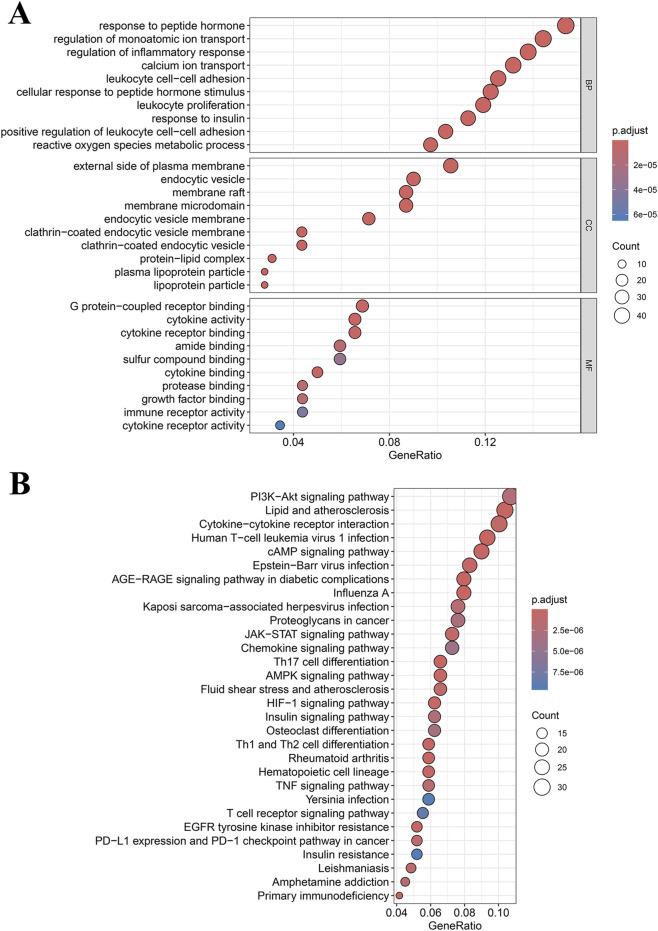
Functional enrichment analysis. **(A)** GO functional enrichment analysis of DEGMGs. **(B)** KEGG pathway enrichment analysis of DEGMGs.

Functional enrichment analysis of DEGMGs indicated significant correlations. For GO terms (Figure 2A), biological processes involved inflammatory response regulation, calcium ion transport, leukocyte proliferation, and reactive oxygen species metabolism. Cellular components included membrane rafts and protein-lipid complexes. Molecular functions centered on cytokine activity and immune receptor activity. KEGG pathway analysis highlighted primary immunodeficiency, tumor necrosis factor (TNF) signaling, Janus kinase-signal transducer and activator of transcription (JAK-STAT), T cell receptor signaling, and phosphatidylinositol 3-kinase-Akt (PI3K-Akt) signaling pathways, among others (Figure 2B).

### 3.3 WGCNA construction and key module selection

**FIGURE 3:**
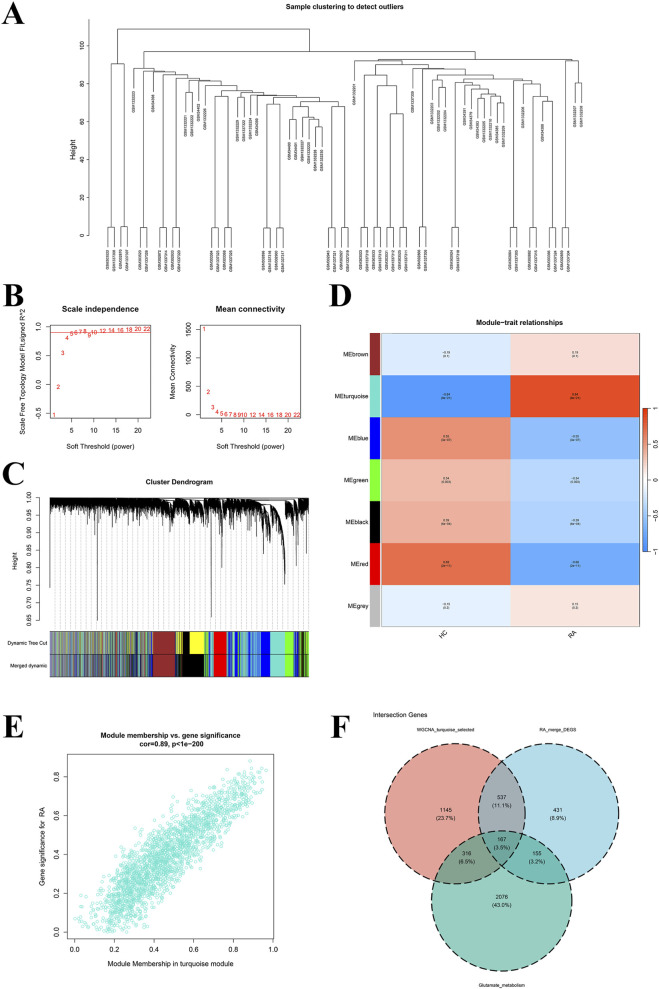
Construction of WGCNA. **(A)** Dendrogram of sample clusters; **(B)** Soft threshold power (left) and average connectivity (right) of WGCNA; **(C)** Gene dendrogram with multiple partitioning modules; different colors represent different clusters (modules); **(D)** Heatmap of correlation between module eigengene (ME) and clinical phenotype; **(E)** Scatter plot of correlation between module membership (MM) and gene significance (GS) of turquoise module; **(F)** Venn diagram of candidate genes.

WGCNA identified gene modules highly correlated with RA clinical traits. Initial sample clustering detected no outliers (Figure 3A). A soft-thresholding power (β) of seven achieved an optimal scale-free topology model fit (Figure 3B). Calculation of TOM and hierarchical clustering, followed by dynamic tree cutting and module merging, yielded seven distinct gene modules (Figure 3C). The turquoise module demonstrated the highest correlation with RA (correlation coefficient = 0.84; Figures 3D,E). Intersecting genes from this module with the DEGs and GMRGs identified 167 candidate genes (Figure 3F).

### 3.4 Machine learning screening of core genes

**FIGURE 4:**
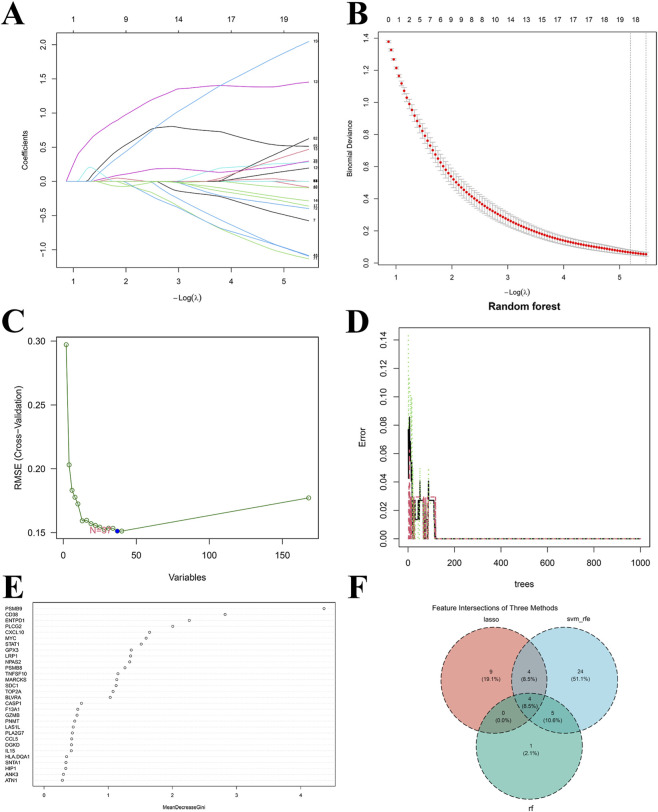
Machine Learning for screening hub genes. **(A)** LASSO regularization path; **(B)** LASSO regression cross-validation curve; **(C)** Line chart of RMSE varying with the number of retained genes, with blue dots indicating the optimal number of genes corresponding to the minimum RMSE; **(D)** The curve of out-of-bag (OOB) error rate varying with the number of decision trees; **(E)** Mean decrease Gini histogram of the top 30 most important genes; **(F)** Venn diagram of the intersection of genes screened by three machine learning algorithms.

Integrating LASSO, SVM-RFE, and RF algorithms refined the selection from the 167 candidate genes. LASSO detected 17 genes (Figures 4A,B). SVM-RFE yielded 37 genes (Figure 4C). RF selected the top 10 genes based on importance (Figures 4D,E). The intersection of these three sets identified CXCL10, ENTPD1, GPX3, and PSMB9 as the core genes (Figure 4F).

### 3.5 Expression analysis and diagnostic potential of core genes

**FIGURE 5:**
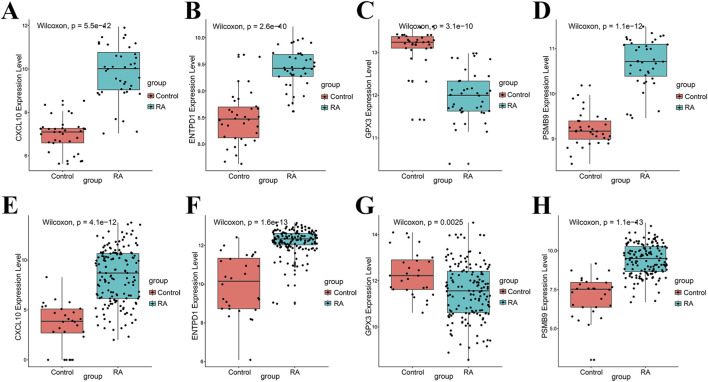
Expression levels of hub genes. **(A)** Expression level of CXCL10 in the training set; **(B)** Expression level of ENTPD1 in the training set; **(C)** Expression level of GPX3 in the training set; **(D)** Expression level of PSMB9 in the training set; **(E)** Expression level of CXCL10 in the validation set; **(F)** Expression level of ENTPD1 in the validation set; **(G)** Expression level of GPX3 in the validation set; **(H)** Expression level of PSMB9 in the validation set.

**FIGURE 6:**
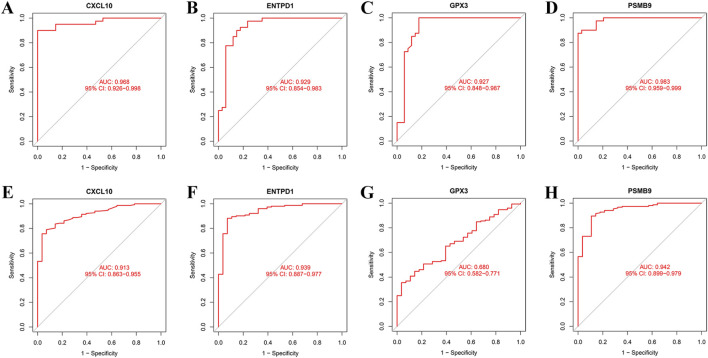
Diagnostic performance of hub genes. **(A)** ROC curve of CXCL10 in the training set; **(B)** ROC curve of ENTPD1 in the training set; **(C)** ROC curve of GPX3 in the training set; **(D)** ROC curve of PSMB9 in the training set; **(E)** The ROC curve of CXCL10 in the validation set; **(F)** The ROC curve of ENTPD1 in the validation set; **(G)** The ROC curve of GPX3 in the validation set; **(H)** The ROC curve of PSMB9 in the validation set.

Boxplot analysis revealed that within the training dataset, expression levels of CXCL10, ENTPD1, and PSMB9 were notably higher in the RA group relative to controls, whereas GPX3 expression was markedly reduced (Figures 5A–D). This expression pattern was fully replicated in the validation dataset (Figures 5E–H). ROC analysis validated the substantial diagnostic potential of these genes for RA. In the training set, all AUCs exceeded 0.9 (AUC_CXCL10_ = 0.968; AUC_ENTPD1_ = 0.929; AUC_GPX3_ = 0.927; AUC_PSMB9_ = 0.983; Figures 6A–D). In the external validation cohort, AUCs for CXCL10, ENTPD1, and PSMB9 remained above 0.9, while the AUC for GPX3 decreased to 0.680 (Figures 6E–H).

### 3.6 GSEA of core genes

**FIGURE 7:**
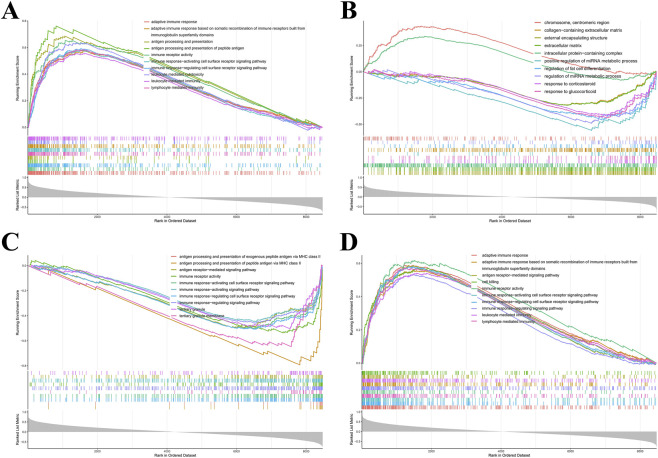
GSEA-GO functional enrichment analysis of hub genes. **(A)** CXCL10; **(B)** ENTPD1; **(C)** GPX3; **(D)** PSMB9.

**FIGURE 8:**
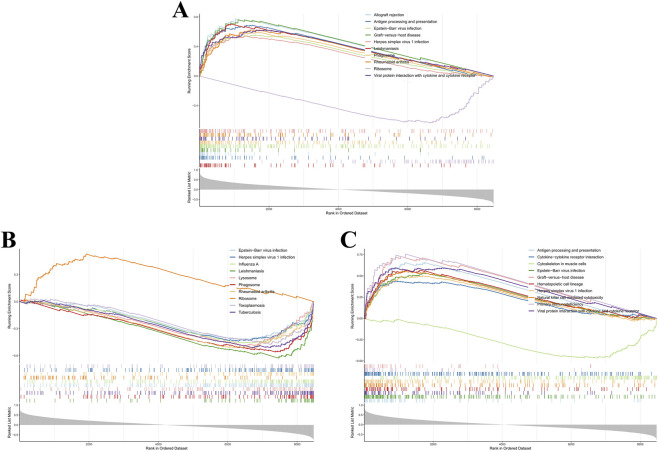
GSEA-KEGG pathway enrichment analysis of hub genes. **(A)** CXCL10; **(B)** GPX3; **(C)** PSMB9.

GSEA elucidated prospective biological processes and signaling cascades associated with the core gene set in RA. GO analysis indicated enrichment for adaptive immune response, leukocyte-mediated immunity, extracellular matrix, regulation of adipocyte differentiation, response to corticosteroid, and cell killing (Figure 7). KEGG analysis revealed significant enrichment in RA, Epstein-Barr virus infection, herpes simplex virus 1 infection, cytokine-cytokine receptor interaction, phagosome, natural killer cell-mediated cytotoxicity, lysosome, and primary immunodeficiency pathways (Figure 8). No significantly enriched KEGG pathways were detected for ENTPD1. These findings suggested that these core genes might have a critical contribution to immune regulation, inflammatory responses, and infection-related processes in RA.

### 3.7 Immune infiltration analysis

**FIGURE 9:**
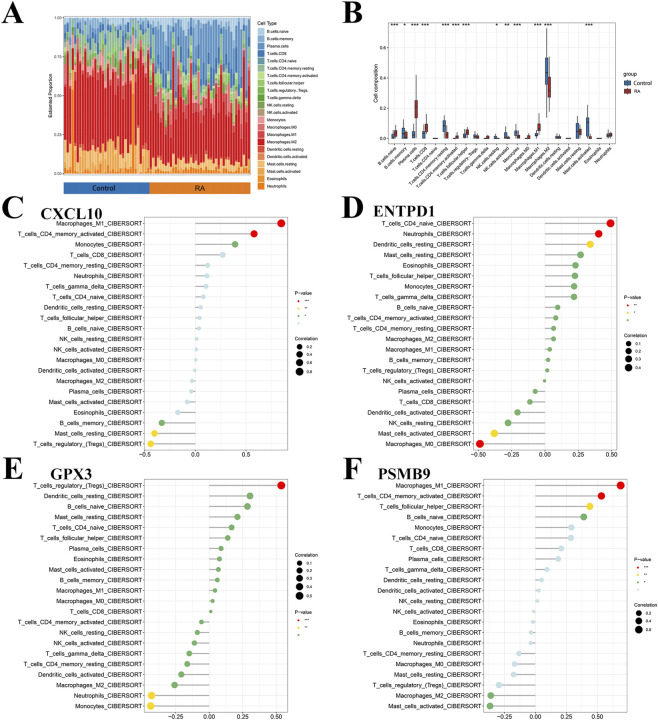
Immune infiltration analysis. **(A)** Stacked bar chart of abundance of immune cells; **(B)** Box plot of differences in immune cell levels between the normal control group and the rheumatoid arthritis group; **(C–F)** Bar graph showing the correlation between the four hub genes and the level of immune cell infiltration. * indicates P < 0.05, ** indicates P < 0.01, *** indicates P < 0.001.

CIBERSORT analysis compared immune cell abundances between the RA and control groups. Results indicated substantial differences in the proportions of various immune cell types (Figures 9A,B). Specifically, the RA group exhibited notably higher abundances of naive B cells, plasma cells, CD8^+^ T cells, activated CD4^+^ memory T cells, follicular helper T cells, and M1 macrophages. Conversely, the RA group showed markedly lower abundances of memory B cells, resting CD4^+^ memory T cells, resting natural killer cells, activated natural killer cells, monocytes, M2 macrophages, and activated mast cells. Subsequent analysis investigated correlations linking core gene expression to immune cell infiltration. Bar plots demonstrated significant correlations of the core genes with multiple immune cell types (Figures 9C–F).

### 3.8 Single-cell sequencing

**FIGURE 10:**
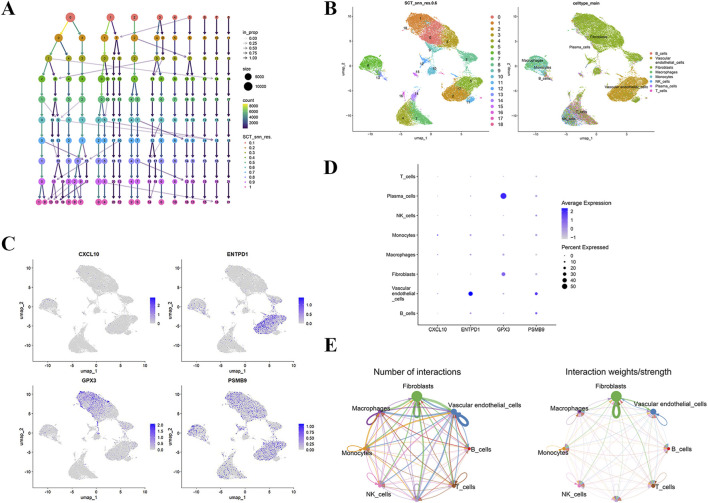
Single-cell sequencing analysis. **(A)** Dendrogram showing clustering results at various resolutions; **(B)** UMAP plot of cell clustering at a resolution of 0.6 (left) and annotation results of major cell types (right); **(C)** Expression distribution of hub genes on UMAP; **(D)** Bubble plot of expression of hub genes in different cell types; **(E)** Chord plot of the number (left) and strength (right) of cell interactions based on ligand-receptor pairs.

Integration of four RA synovial tissue specimens from the GSE200815 dataset, following stringent quality control, retained 33,188 cells and 21,180 genes for analysis. Dimensionality reduction and clustering partitioned cells into 18 clusters (Figure 10A). Annotation based on marker genes and literature identified eight major cell types: B cells (markers: CD79 A/B, MS4A1, CD19) (Guo et al., 2024; Jiao et al., 2024; Sun et al., 2025a), T cells (CD3D, CD4, IL7R, CD8A, GZMK) (Andreatta et al., 2021; Li et al., 2024b; Liu et al., 2022), vascular endothelial cells (PECAM1, VWF, CLDN5, ENG, CDH5) (Fu and Song, 2021; Li et al., 2025; Qiu et al., 2025), fibroblasts (DCN, LUM, COL1A1, COL3A1, DPT, FAP, THY1, ITGA5, POSTN) (Kemble and Croft, 2021; Micheroli et al., 2022), macrophages (CD163, C1QA/B/C, SPP1, FCGR3A) (Murthy et al., 2022; Thornton et al., 2019), monocytes (FCN1, S100A8/9, VCAN, LYZ) (Liao et al., 2020; Tang-Huau et al., 2018), natural killer cells (NKG7, GNLY) (Rebuffet et al., 2024), and plasma cells (JCHAIN, MZB1, IGHG1, XBP1, IGLC2) (Fan et al., 2024; Wen et al., 2020) (Figure 10B). Analysis of gene expression patterns revealed high ENTPD1 expression in vascular endothelial cells, elevated GPX3 expression in fibroblasts and plasma cells, and prominent PSMB9 expression in vascular endothelial and B cells (Figures 10C,D). Cell-cell interaction analysis indicated numerous and strong interactions between fibroblasts and vascular endothelial cells (Figure 10E). These results provided a foundation for investigating core gene regulation within key cell types and deciphering the cellular communication network in the RA synovial microenvironment.

### 3.9 Drug prediction and molecular docking

**FIGURE 11:**
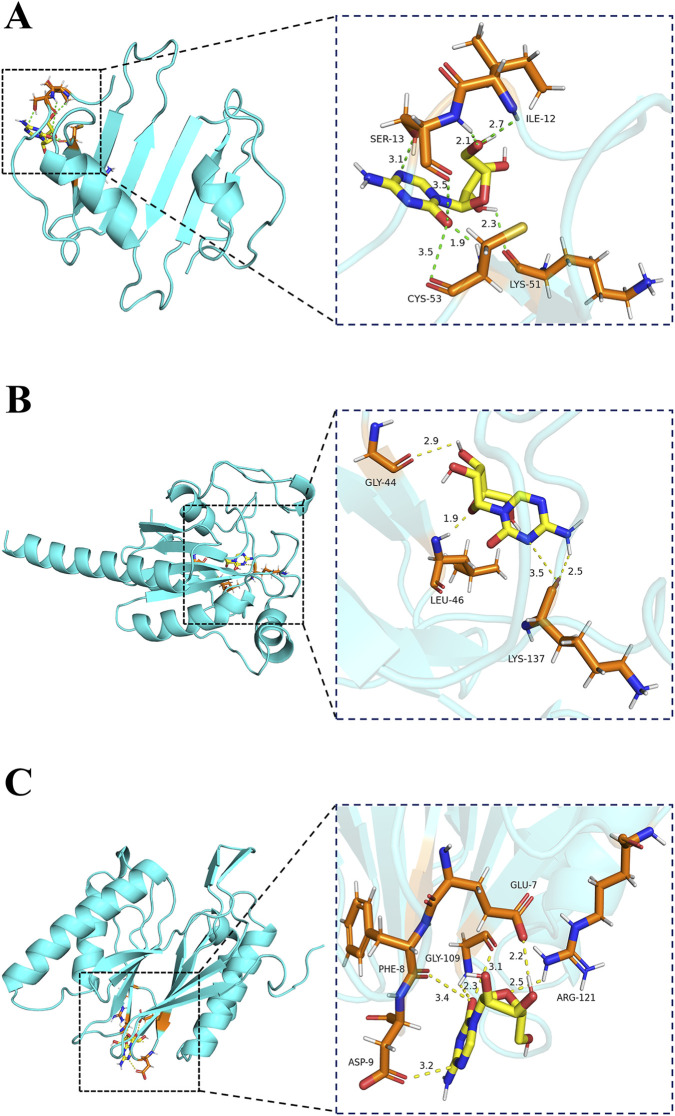
Molecular docking. **(A)** Molecular docking of CXCL10 with azacitidine; **(B)** Molecular docking of GPX3 with azacitidine; **(C)** Molecular docking of PSMB9 with azacitidine.

We input the top 150 upregulated and top 150 downregulated DEGMGs (Supplementary Table S2), ranked by absolute log2FC, into the CMap database for compound prediction. Finally, the 10 compounds with the lowest scores were selected as core candidate therapeutic molecules, including cephaeline, digitoxin, emetine, ouabain, narciclasine, azacitidine, HU-211, verrucarin A, cycloheximide, and anisomycin (Supplementary Table S3). Azacitidine, a DNA methyltransferase inhibitor, can remodel gene expression via demethylation (Landman et al., 2021). Studies confirm its ability to induce FOXP3 promoter demethylation *in vitro* (Sánchez-Abarca et al., 2010). This action upregulated FOXP3 expression, expanded functional Tregs, and exerted anti-inflammatory effects (Nakano et al., 2013). Work by Petralia and Tóth further reported that azacitidine suppressed key inflammatory factors like interleukin (IL)-6 and TNF-α in RA mouse models (Petralia et al., 2019; Tóth et al., 2019). This suppression significantly alleviated joint symptoms and delayed RA progression. To further validate the binding capacity of azacitidine with the core target protein, a global blind docking analysis was performed across the entire protein surface. The algorithm autonomously searched for and identified the pocket region with the most favorable binding energy. This provided an objective and unbiased prediction of potential interaction modes between the small molecule and the target protein. Docking scores (kcal/mol) for azacitidine with CXCL10, GPX3, and PSMB9 were −5.2, −5.8, and −5.7, respectively (Figures 11A–C). All complexes exhibited binding energies lower than −5 kcal/mol and formed multiple stable intermolecular interactions, suggesting that azacitidine can bind spontaneously to the aforementioned target proteins with favorable affinity, among which the binding to GPX3 was the strongest. These results indicate that CXCL10, GPX3, and PSMB9 may serve as potential targets of azacitidine in RA.

### 3.10 Molecular dynamics simulation

**FIGURE 12:**
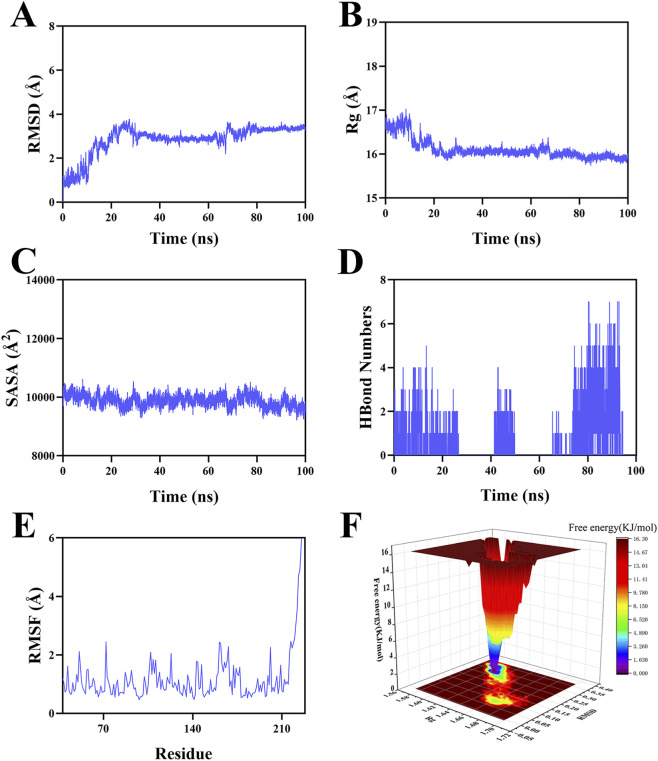
Molecular dynamics simulation. **(A)** RMSD values of the GPX3-azacitidine complex over time; **(B)** Rg values of the GPX3-azacitidine complex over time; **(C)** SASA values of the GPX3-azacitidine complex over time; **(D)** Number of HBonds in the GPX3-azacitidine complex over time; **(E)** RMSF values of the GPX3-azacitidine complex; **(F)** Landscape of free energy.

To assess the stability of the GPX3-azacitidine complex, a molecular dynamics simulation was performed over a 100-ns period. The RMSD, a key metric for conformational stability, indicated that the system reached equilibrium after ∼70 ns, stabilizing around 3.3 Å (Figure 12A). This suggested overall conformational stability. The Rg value converged from an initial ∼16.7 Å to a stable ∼16 Å, indicating a compact and robust complex structure (Figure 12B). The stable SASA profile further supported conformational stability (Figure 12C). Hydrogen bonds between the ligand and protein ranged from zero to seven throughout the trajectory, averaging around two (Figure 12D). This contributed significantly to complex stability. Low RMSF values (<2 Å for most of the simulation) indicated low local residue flexibility and high structural stability (Figure 12E). Based on RMSD and Rg values, the free energy landscape (color gradient: red for high energy, blue for low energy) illustrated the energy distribution during the simulation (Figure 12F). These results collectively demonstrated the kinetic stability of the azacitidine-GPX3 interaction.

### 3.11 Experimental validation of core genes

**FIGURE 13:**
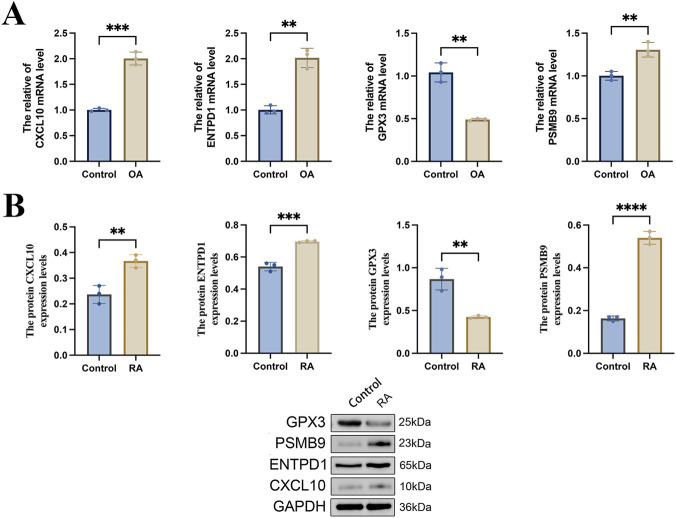
Validation of expression of hub genes. **(A)** qRT-PCR for detecting the mRNA expression levels of CXCL10, ENTPD1, GPX3, and PSMB9 in the cell model (n = 3); **(B)** Western blot (WB) for detecting the protein expression levels of CXCL10, ENTPD1, GPX3, and PSMB9 in the cell model (n = 3). * denotes p < 0.05, ** denotes p < 0.01, *** denotes p < 0.005, **** denotes p < 0.001.

qRT-PCR and WB experiments validated core gene expression in a human RA FLS model. qRT-PCR confirmed that mRNA expression of CXCL10, ENTPD1, and PSMB9 was notably elevated, whereas that of GPX3 was reduced, in the RA group compared to controls (Figure 13A). Similarly, WB analysis showed consistent changes at the protein level, with elevated levels of CXCL10, ENTPD1, and PSMB9 and a reduction in GPX3 (Figure 13B). Complete WB band information is provided in Supplementary Figure S1. These results strongly corroborated the bioinformatics predictions, confirming the differential expression signature of the core genes.

## 4 Discussion

RA is a chronic inflammatory arthropathy marked by symmetric joint swelling, pain, and morning stiffness. It originates from autoimmune dysregulation that is closely linked to genetic and environmental factors (Giannini et al., 2020). Without early intervention, persistent inflammation can cause joint deformity and functional loss (Monti et al., 2015). This underscores the critical importance of early diagnosis and treat-to-target strategies. Recent investigations indicate that glutamate metabolism may contribute to RA pathology by modulating inflammatory responses, immune balance, energy supply, and bone metabolism (Cowan et al., 2012; Flood et al., 2007; Gangadharan et al., 2011; Hajati et al., 2009; Hanlon et al., 2022; Kim et al., 2014; Matias et al., 2021; Wen et al., 2025). However, its precise mechanistic roles and clinical value as a biomarker require further elucidation. The current investigation integrated bioinformatics and machine learning to systematically detect possible glutamate metabolism-related biomarkers and candidate drugs. The goal was to provide new insights for the diagnosis and targeted therapy of RA.

Enrichment analyses demonstrated that DEGMGs were notably enriched in classic RA immune-inflammatory pathways, including TNF, JAK-STAT, and PI3K-Akt signaling. The TNF signaling pathway acts as a core initiator of the inflammatory cascade in RA (Bradley, 2008). Its aberrant activation amplifies the pro-inflammatory effects of synovial cells by regulating key cytokines like TNF-α, IL-1, and IL-6, thereby accelerating inflammatory infiltration and tissue damage (Elshabrawy et al., 2015; Jang et al., 2021). Several TNF-α monoclonal antibodies are now approved for RA treatment, significantly reducing synovitis and joint symptoms (Atzeni et al., 2021; Xiao et al., 2025). Recent evidence confirms the involvement of aberrant JAK-STAT signaling in RA pathogenesis (Ding et al., 2023; Riitano et al., 2023). As a central hub for immune signal transduction, this pathway regulates the differentiation and function of key immune cells like T cells and macrophages via STAT protein phosphorylation, exacerbating immune imbalance and driving RA advancement (Bousoik and Montazeri Aliabadi, 2018; Ciobanu et al., 2020; Malemud, 2013). Furthermore, aberrant PI3K-Akt pathway activation is another critical event in RA pathology, aggravating joint inflammation and tissue damage by modulating inflammatory molecule expression, FLS proliferation, and angiogenesis (Ba et al., 2021; Dinesh and Rasool, 2018; Mitra et al., 2012; Tsai et al., 2017; Zou et al., 2016). These results imply that DEGMGs might be deeply involved in RA immune-inflammatory responses and pathology through cross-talk between multiple pathways. This provides a theoretical basis for developing novel RA treatments by targeting these pathways.

Machine learning identified four key GMRGs: CXCL10, ENTPD1, GPX3, and PSMB9. CXCL10 is a pivotal inflammatory chemokine in RA pathogenesis (Dillemans et al., 2023). Binding specifically to its receptor CXCR3, CXCL10 recruits immune cells like T cells and monocytes to the synovium (Rokni et al., 2025). This exacerbates local inflammation and sustains synovitis. Studies confirm significantly elevated levels of CXCL10 in the serum and synovial fluid of untreated RA patients, correlating with disease activity (Imam et al., 2019; Kuan et al., 2010). This suggests its potential as an early diagnostic biomarker (Pandya et al., 2017). Regarding the regulation of bone metabolism, CXCL10 can also facilitate osteoclastogenesis by enhancing the expression of RANKL in CD4^+^ T lymphocytes, thereby participating in RA-related bone destruction and playing a potential role in the progression of joint erosion (Lee et al., 2011; Lee et al., 2017). Furthermore, pro-inflammatory factors within the inflammatory microenvironment can induce the release of cytotoxic molecules such as glutamate, and the aberrant accumulation of glutamate can suppress the formation of bone (Chenu et al., 1998; Cowan et al., 2012), thereby exacerbating bone loss—a key pathological feature of RA (Sakthiswary et al., 2022). More importantly, existing research indicates that the reprogramming of glutamate metabolism induced by the deficiency of MTAP can directly suppress the expression of CXCL10 (Chang et al., 2025). There may be a functional linkage between CXCL10 and glutamate metabolism, implicating both in the regulation of the immune microenvironment. This opens a new avenue for exploring the pathophysiological mechanisms of RA. ENTPD1 (CD39) is an ectonucleoside triphosphate diphosphohydrolase 1 and hydrolyzes excess pro-inflammatory extracellular adenosine triphosphate and adenosine diphosphate to adenosine monophosphate (Almada et al., 2025). This provides the substrate for CD73-mediated adenosine production (Ortiz et al., 2023). The produced adenosine exerts anti-inflammatory effects by suppressing immune cell activation and pro-inflammatory cytokine release, thereby curbing excessive joint inflammation (Shen et al., 2025). In autoimmune diseases, CD39 holds significant biomarker potential. Its expression level not only correlates closely with disease activity but also carries important value in predicting treatment response (Shen et al., 2025). Furthermore, single-cell sequencing analysis revealed high ENTPD1 expression specifically in vascular endothelial cells. This finding is closely associated with the synovial pannus—a pathological hallmark of RA whose formation and development are central to joint tissue damage (Wu et al., 2025). Evidence confirms that the ENTPD1-mediated adenosine signaling pathway directly regulates the proliferation, migration, and tube-forming capacity of vascular endothelial cells (Jackson et al., 2007; Kanthi et al., 2015). This process thereby potentially promotes neovascularization (Kauffenstein et al., 2010) and drives the formation and progression of the invasive synovial pannus. GPX3 encodes glutathione peroxidase 3 and is a key antioxidant enzyme in the body. Its catalytic function is highly dependent on glutathione, which is synthesized from glutamate as a precursor (Chang et al., 2020; Song et al., 2024). The inflammatory RA microenvironment generates abundant reactive oxygen species (Zhang et al., 2024). GPX3 helps maintain redox balance by scavenging reactive oxygen species, mitigating oxidative stress-induced joint damage (Zhang et al., 2024). The expression of GPX3 in synovial fibroblasts is also associated with disease progression and pain (Nanus et al., 2021). Notably, Liang et al. also identified GPX3 as a potential RA biomarker, consistent with our findings (Liang et al., 2025). PSMB9, a component of the immunoproteasome, is crucial for antigen processing and presentation (Mufti et al., 2021). Growing evidence links it to autoimmune diseases (Casp et al., 2003; Li et al., 2024a; Nakamura et al., 2016; Teisserenc et al., 1997; Zeng et al., 2023). Liu et al. demonstrated that PSMB9 inhibitors suppress inflammation and proliferation in synovial cells (Liu et al., 2025). This provides experimental support for PSMB9-targeted RA therapies.

Regarding diagnostic performance, the AUC for GPX3 decreased from 0.927 in the training cohort to 0.680 in the external validation cohort. A comparison of baseline characteristics between the two cohorts revealed marked disparities in age distribution of patients, stage of diseases, prior medication history, criteria for inclusion of samples, and detection platforms (Guo et al., 2017; Huber et al., 2008; Walsh et al., 2017; Woetzel et al., 2014). Each of these factors could contribute to variability in the diagnostic performance of a single biomarker across different cohorts. The modest decline in the diagnostic efficacy of GPX3 in the external validation set likely reflects the profound heterogeneity and complex pathogenesis of RA. This does not imply that GPX3 has lost its value as a biomarker. An AUC around 0.7 suggests that a marker possesses certain diagnostic value (Sun et al., 2025b; Xu et al., 2025). With an AUC of 0.680 in the current research, the performance of GPX3 is highly proximate to this threshold, further indicating its potential clinical relevance. In contrast, CXCL10, ENTPD1, and PSMB9 demonstrated excellent and stable diagnostic efficacy (AUC >0.9) in both the training and external validation cohorts. This indicates their strong potential for clinical application. Although the AUC for GPX3 was slightly lower in external validation, it exhibited a consistently stable trend of differential expression across datasets and *in vitro* cellular experiments. Furthermore, it plays an irreplaceable and significant biological role in the injury of oxidative stress associated with RA. In summary, these four hub genes participate in distinct pathological processes of RA, each offering unique advantages and complementing the others. Their combined application could more comprehensively reflect the characteristics of the disease, thereby enhancing the accuracy and stability of the diagnosis of RA.

The pathological progression of RA is closely linked to immune infiltration. The synovium is the primary lesion site and attracts massive immune cell infiltration, provoking persistent inflammation. Immune infiltration analysis revealed significant differences in T cells, B cells, plasma cells, and macrophages between RA and healthy synovial tissues (Ding et al., 2023). Notably, activated CD4^+^ memory T cells and T follicular helper cells were markedly upregulated in RA synovium compared to normal tissue. CD4^+^ Th17 cells were aberrantly activated, secreting pro-inflammatory cytokines (Kondo et al., 2018). These cytokines recruit neutrophils and monocytes, thereby amplifying local inflammation and disrupting immune balance (Tu et al., 2021). Mature T follicular helper cells promote the differentiation of B cells into plasma cells, which secrete autoantibodies, such as rheumatoid factor, via signaling pathways involving IL-21, CD40L, and ICOS (Lu et al., 2021). These autoantibodies form immune complexes that activate the complement system (Song et al., 2024). B cells can also interact with CD4^+^ T cells by presenting antigens, thereby promoting the release of pro-inflammatory cytokines (Nandakumar et al., 2023; Riitano et al., 2025). Macrophages polarize into pro-inflammatory (M1) and anti-inflammatory (M2) phenotypes. RA synovium showed a significantly increased M1/M2 ratio. M1 macrophages secrete pro-inflammatory mediators, exacerbating synovitis and tissue damage, while anti-inflammatory M2 macrophage function is relatively impaired (Huang et al., 2021a; Udalova et al., 2016). Furthermore, significant correlations were observed between hub gene expression and infiltration of specific immune cells, consistent with their involvement in inflammatory responses. Investigating the regulatory mechanisms of these differential immune cells offers new directions for precise RA intervention.

Finally, azacitidine was predicted as a potential therapeutic small-molecule drug for RA using the CMap database. Molecular docking and molecular dynamics simulations verified its favorable binding affinity and stability with the target protein. Azacitidine is an approved epigenetic modulator for hematologic malignancies that primarily functions by inhibiting DNA methyltransferase activity and inducing target gene demethylation (Tran et al., 2011). It exerts multifaceted biological effects by influencing the activation of inflammation-related pathways and the functional differentiation of immune cells (Daskalakis et al., 2002). GPX3 represents a critical antioxidant enzyme. The expression and activity of GPX3 are frequently suppressed in various inflammatory diseases due to hypermethylation of its promoter region, subsequently exacerbating the damage of oxidative stress (Mohamed et al., 2014; Ye et al., 2025; Zhang et al., 2021). Research has confirmed that hypermethylation of CpG islands in the promoter of GPX3 promotes chondrocyte apoptosis in patients with Kashin-Beck disease (Zhang et al., 2022). This suggests a critical role for aberrant methylation of GPX3 in the progression of osteoarthropathy. RA, a prototypical autoimmune inflammatory disease, is characterized by disruptions in the methylation patterns of DNA during its development. Thus, we hypothesize that azacitidine may ameliorate the aberrant hypermethylation of the promoter of GPX3 in patients with RA to restore the normal expression and antioxidant function of GPX3. This modulates inflammatory responses and immune balance at an epigenetic level, ultimately exerting a potential therapeutic effect on RA (Huang et al., 2021b). Fu et al. demonstrated that treating peripheral blood mononuclear cells from RA patients with azacitidine led to significant demethylation of hypermethylated CpG motifs in upstream genes (Fu et al., 2011). This substantially increased the mRNA and protein expression levels of the immunosuppressive cytokine IL-10 and inhibited RA progression. However, the inherent cytotoxicity and systemic effects of azacitidine must be acknowledged. Future research will focus on a core “low-dose epigenetic priming” strategy to enable its safe application in RA therapy (McCormack and Warlick, 2010). This strategy involves optimizing individualized dosing schedules, developing targeted delivery systems, and exploring combination therapies. The goal is to systematically evaluate target specificity, administration safety, and long-term efficacy, ultimately confining dose-limiting toxicities to a manageable and reversible range.

The current research has its limitations. Primarily based on bioinformatics and preliminary cellular validation, the conclusions require validation using human tissues, various arthritis animal models, or different cell lines. Future work should include systematic *in vitro* and *in vivo* functional experiments to elucidate the mechanistic roles of the core genes, alongside multi-center, large-scale cohort data for further validation. Secondly, experimental evidence for azacitidine in treating RA is limited, and its precise mechanisms are not fully elucidated. Subsequent targeted pharmacological studies are needed to explore its molecular actions in RA, supporting clinical translation. The immune infiltration analysis was derived solely from computational inferences using the CIBERSORT algorithm and has not been directly validated through experimental techniques such as flow cytometry. This may reduce the reliability of the constructed immune microenvironment profile and diminish the explanatory power of the underlying mechanisms. Therefore, future research should employ experimental methods to directly quantify and validate the infiltration levels of immune cells in clinical samples from patients with RA. Pending further confirmatory experimental and clinical data, the general applicability and clinical translational value of these findings remain exploratory, requiring future validation and refinement.

## 5 Conclusion

By integrating bioinformatics analysis with multiple machine learning algorithms, this study identified four GMRGs: CXCL10, ENTPD1, GPX3, and PSMB9. These genes represent potential biomarkers for RA, offering new insights for its molecular diagnosis and targeted therapy. Furthermore, through molecular docking and molecular dynamics simulations, the study predicted azacitidine as a small-molecule drug with potential therapeutic value for RA. Collectively, these findings provide an important foundation for future drug development and clinical research in RA.

## Data Availability

The datasets presented in this study can be found in online repositories. The names of the repository/repositories and accession number(s) can be found in the article/Supplementary Material.
