# Supplementary figures and images for: Integrating multi-omics, machine learning, and molecular dynamics simulations to identify glutamate metabolism-related biomarkers and drug candidates in rheumatoid arthritis
Source: Front Mol Biosci. 2026 Apr 29;13:1834429. doi: 10.3389/fmolb.2026.1834429 (PMC13167439; doi:10.3389/fmolb.2026.1834429)

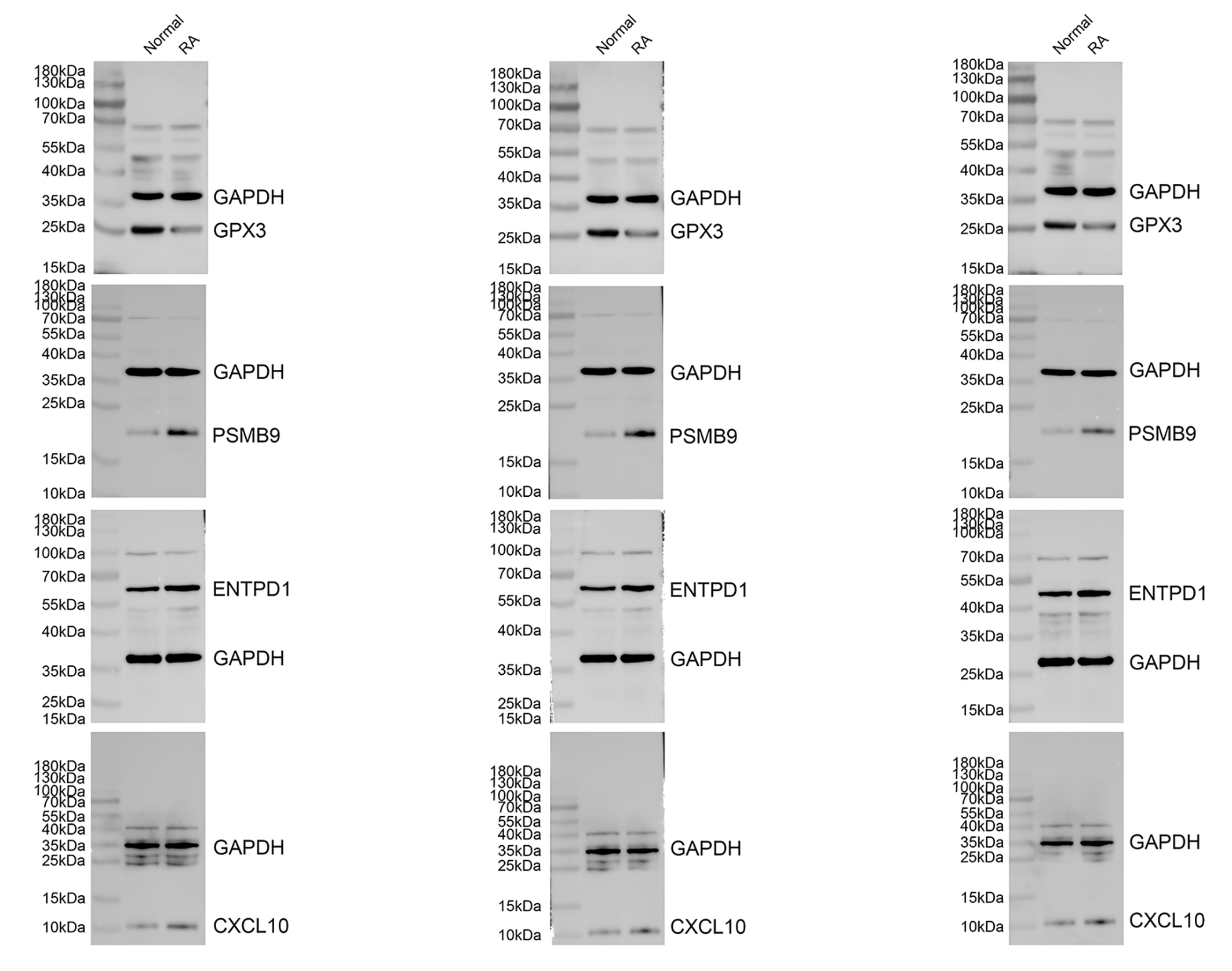

Supplement: Supplementary file 3 [file Image1.tif]
